# Supplementary material for: The widespread nature of Pack-TYPE transposons reveals their importance for plant genome evolution
Source: PLoS Genet. 2022 Feb 24;18(2):e1010078. doi: 10.1371/journal.pgen.1010078 (PMC8903248; doi:10.1371/journal.pgen.1010078)
Supplement: S1 Fig — (PDF) [file pgen.1010078.s001.pdf]

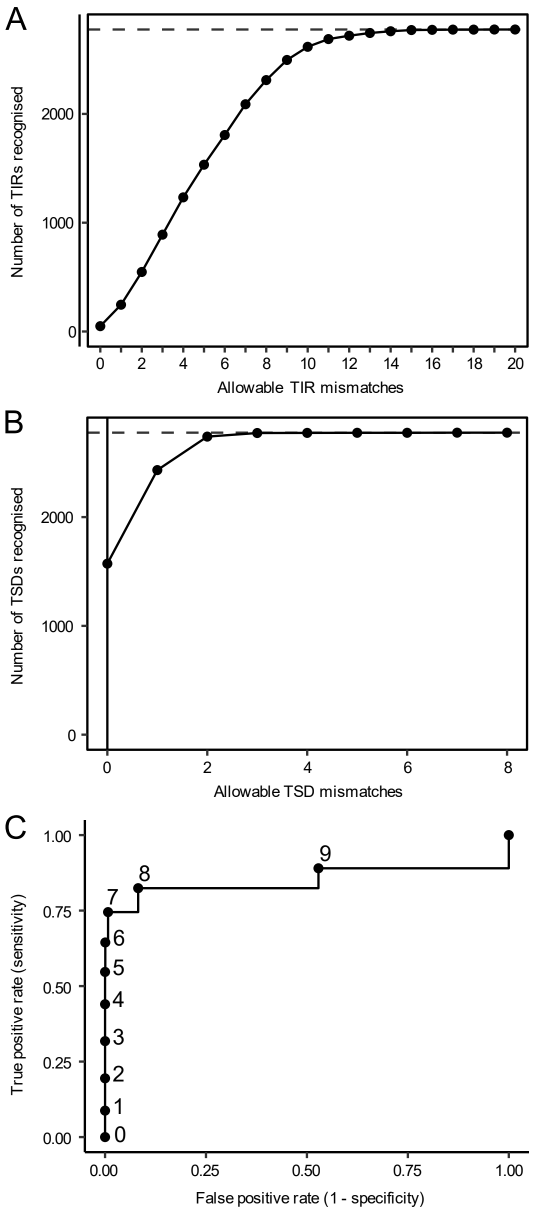

S1 Fig. Benchmarking *packFinder* against previously annotated Pack-MULEs. The performance of *packFinder* applied to a set of 2776 TIR-based Pack-MULEs identified previously [1]. **A** The number of Pack-MULE TIRs recognised by *packFinder* as a function of the allowable TIR mismatch parameter. **B** The number of Pack-MULE TSDs recognised by *packFinder* as a function of the allowable TSD mismatch parameter. **C** A receiver operating characteristic (ROC) curve demonstrating the overall performance of *packFinder*. The number of allowable TIR mismatches was varied, while the number of TSD mismatches was held constant at 2. The true-positive rate (sensitivity) was calculated as the proportion of the 2776 Pack-MULEs identified by *packFinder*; we then predicted the number of false-positive annotations made by *packFinder* (see **Methods**) to estimate the false-positive rate.

Reference:

1. Jiang N, Bao Z, Zhang X, Eddy SR, Wessler SR. Pack-MULE transposable elements mediate gene evolution in plants. Nature. 2004;431: 569–573. doi:10.1038/nature02953
