# Supplementary material for: The widespread nature of Pack-TYPE transposons reveals their importance for plant genome evolution
Source: PLoS Genet. 2022 Feb 24;18(2):e1010078. doi: 10.1371/journal.pgen.1010078 (PMC8903248; doi:10.1371/journal.pgen.1010078)
Supplement: S3 Fig — (PDF) [file pgen.1010078.s003.pdf]

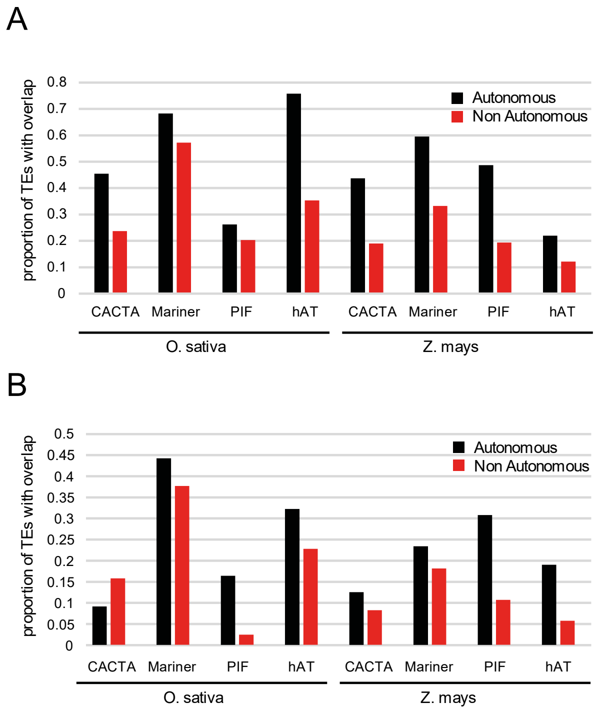

S3 Fig. Annotation of autonomous and non-autonomous TIR TEs. Bar plots showing the matches of the public TE annotation available for the rice (IRGSP1) and maize (B73) genomes, and the functional annotation produced by *packFinder*. **A** Proportion of *packFinder*-annotated TEs that overlap (>1 nt) at least one reference-annotated feature classified as belonging to the same superfamily. Data are displayed separately for TEs classified by *packFinder* as Autonomous or Non-Autonomous (non-Pack + Pack-TYPE). TEs classified simply as MITEs were considered a good match for both *Mariner* and *PIF* elements in rice. **B** Proportion of *packFinder*-annotated TEs with more than 50% of their sequence overlapping reference-annotated features classified as belonging to the same superfamily. Description as in **A**.
