# Supplementary material for: The widespread nature of Pack-TYPE transposons reveals their importance for plant genome evolution
Source: PLoS Genet. 2022 Feb 24;18(2):e1010078. doi: 10.1371/journal.pgen.1010078 (PMC8903248; doi:10.1371/journal.pgen.1010078)
Supplement: S4 Fig — (PDF) [file pgen.1010078.s004.pdf]

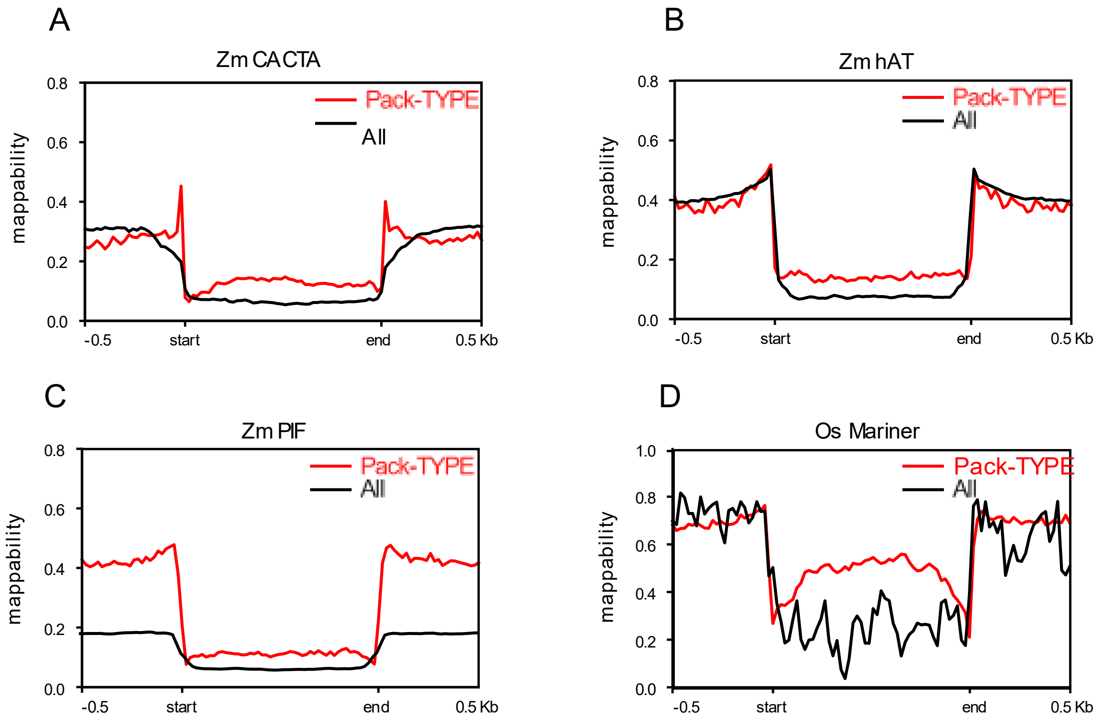

S4 Fig. Repetitiveness of annotated Pack-TYPE-TEs. Distribution of averaged uniqueness of DNA sequence (mappability, see **Methods**), calculated for Pack-TYPE elements belonging to *CACTA* (**A**), *hAT* (**B**) and *PIF* (**C**) superfamilies in maize and for Pack-Mariner (**D**) in rice. The average mappability of all annotated elements belonging to each superfamily in the relevant genome (marked with “All”) have been plotted for comparison.
