# Supplementary material for: The widespread nature of Pack-TYPE transposons reveals their importance for plant genome evolution
Source: PLoS Genet. 2022 Feb 24;18(2):e1010078. doi: 10.1371/journal.pgen.1010078 (PMC8903248; doi:10.1371/journal.pgen.1010078)
Supplement: S5 Fig — (PDF) [file pgen.1010078.s005.pdf]

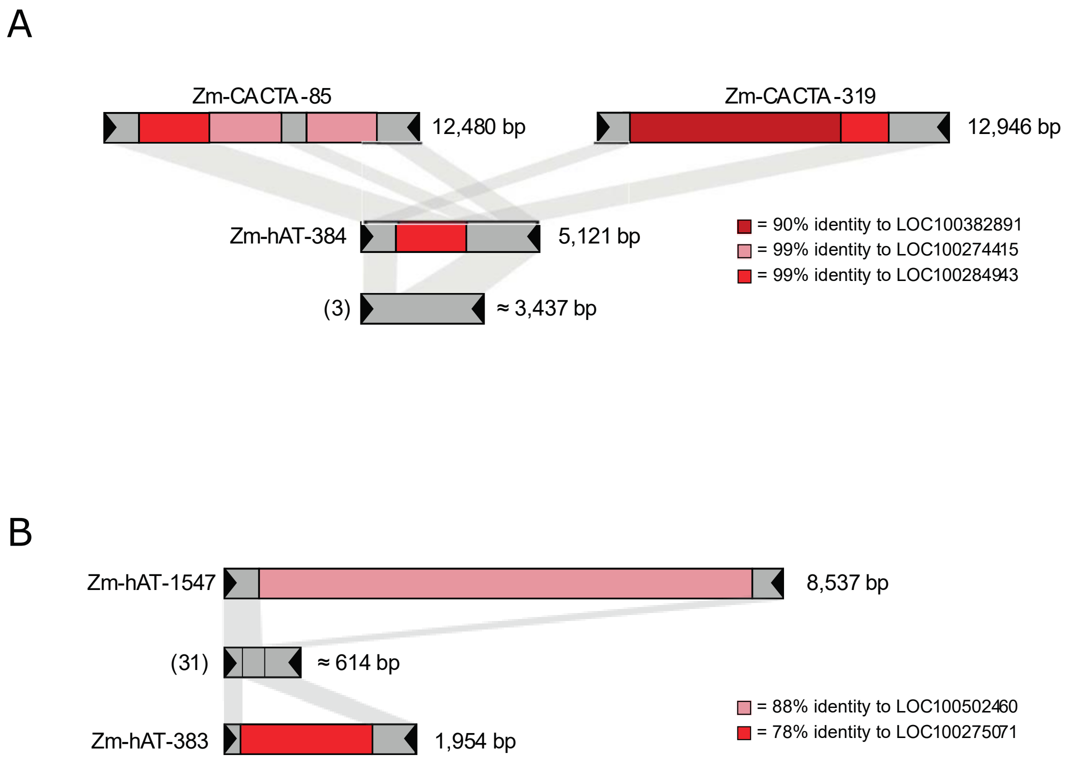

S5 Fig. Examples of sequence structure for Pack-CACTA and Pack-hAT TEs. Each bar summarises the structure of a group (with number of elements displayed in brackets) or single elements. Shaded grey marks regions with sequence homology. Element sizes are displayed on the right. Regions in different shades of red mark DNA sequences containing homology to coding genes. The black triangles mark the TIRs (15 nt). The panels show the structure of: **A** Zm-Pack-CACTA TE family #13; **B** Zm-Pack-hAT TE family #14.
