# Supplementary material for: The widespread nature of Pack-TYPE transposons reveals their importance for plant genome evolution
Source: PLoS Genet. 2022 Feb 24;18(2):e1010078. doi: 10.1371/journal.pgen.1010078 (PMC8903248; doi:10.1371/journal.pgen.1010078)
Supplement: S6 Fig — (PDF) [file pgen.1010078.s006.pdf]

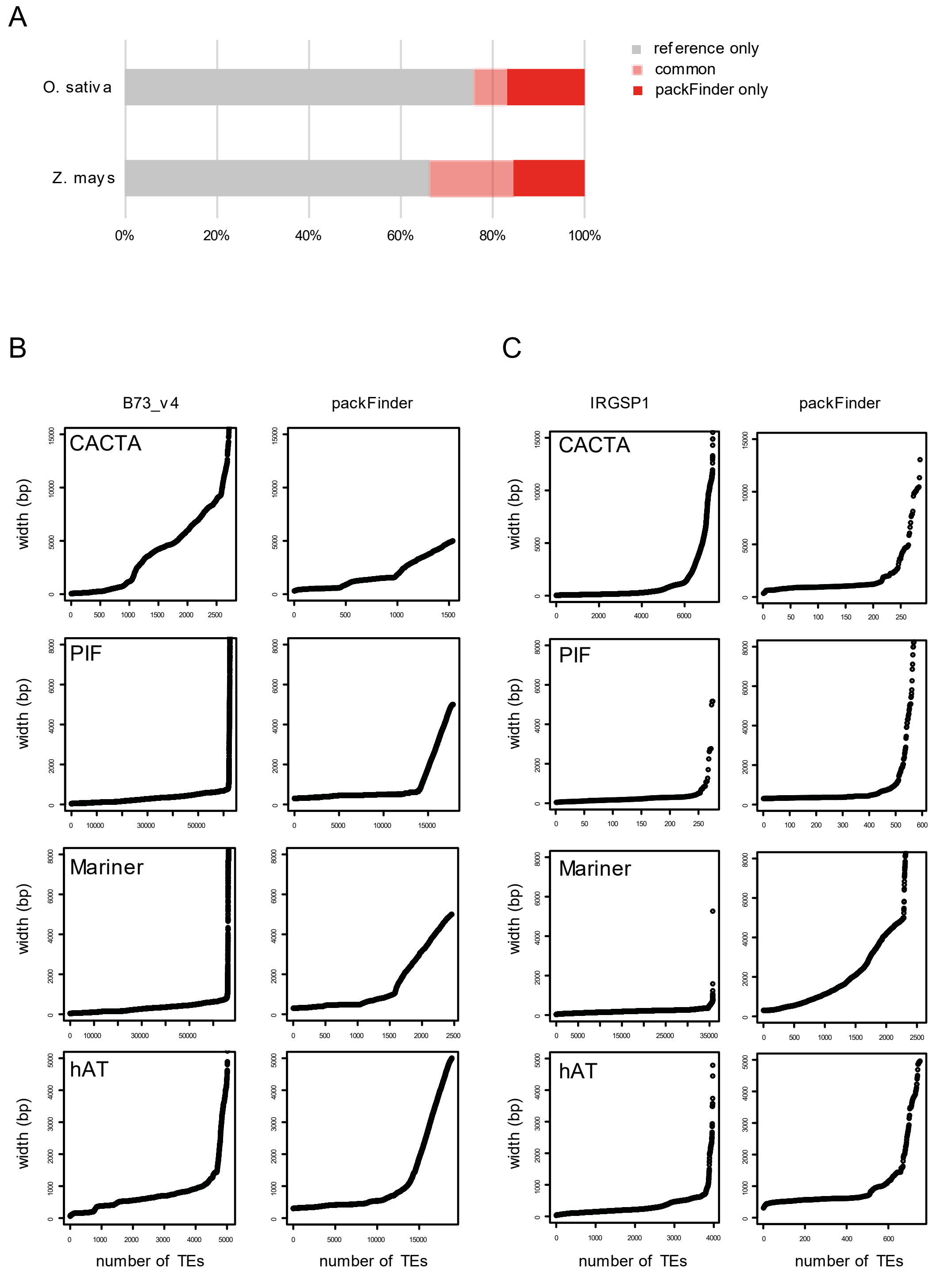

S6 Fig. Comparison of *packFinder* and reference TE annotations. A Proportion of TEs found by *packFinder* in the maize genome compared to the rice (IRGSP1) and maize (B73_v4) TE annotations for analysed TIR TE superfamilies. Each bar shows TEs found only in the reference annotations, only by *packFinder*, or present in both lists (common). Note that, due to the high number of small TE fragments annotated in the *O. sativa* genome, only elements >300 bp were considered. **B** Ordered widths of annotated (B73) and *packFinder*-screened TEs in maize for each superfamily analysed. **C** Ordered widths of annotated (IRGSP1) and *packFinder*-screened TEs in rice for each superfamily analysed. Elements annotated as "MITE" were included in the *Mariner* group.
