# Supplementary material for: The widespread nature of Pack-TYPE transposons reveals their importance for plant genome evolution
Source: PLoS Genet. 2022 Feb 24;18(2):e1010078. doi: 10.1371/journal.pgen.1010078 (PMC8903248; doi:10.1371/journal.pgen.1010078)
Supplement: S7 Fig — (PDF) [file pgen.1010078.s007.pdf]

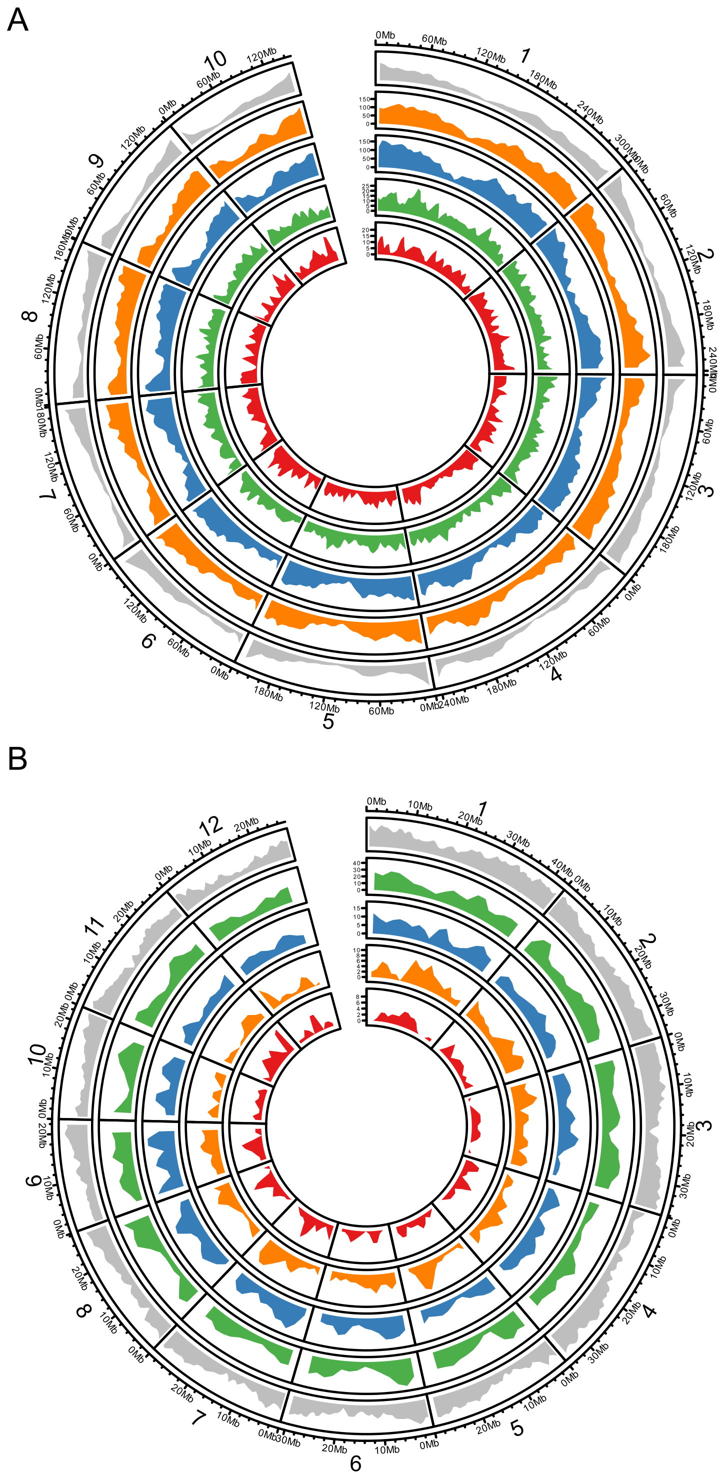

S7 Fig. The whole-genome distribution of TEs annotated using TIRs derived from TIR Learner. Density plots generated using Circos [1] for the distribution of the annotated TEs in *Zea mays* (**A**) and *Oryza sativa* (**B**) genomes. TE superfamilies are coloured and separated by track, ordered by their relative abundance in the genome. The distribution of genes (in grey) is plotted for comparison. The outer track displays genomic positions while the horizontal axis refers to TE frequency.

Reference:

1. Krzywinski MI, Schein JE, Birol I, Connors J, Gascoyne R, Horsman D, et al. Circos: An information aesthetic for comparative genomics. Genome Res. 2009 [cited 16 Dec 2021]. doi:10.1101/gr.092759.109
