# Supplementary material for: The widespread nature of Pack-TYPE transposons reveals their importance for plant genome evolution
Source: PLoS Genet. 2022 Feb 24;18(2):e1010078. doi: 10.1371/journal.pgen.1010078 (PMC8903248; doi:10.1371/journal.pgen.1010078)
Supplement: S8 Fig — (PDF) [file pgen.1010078.s008.pdf]

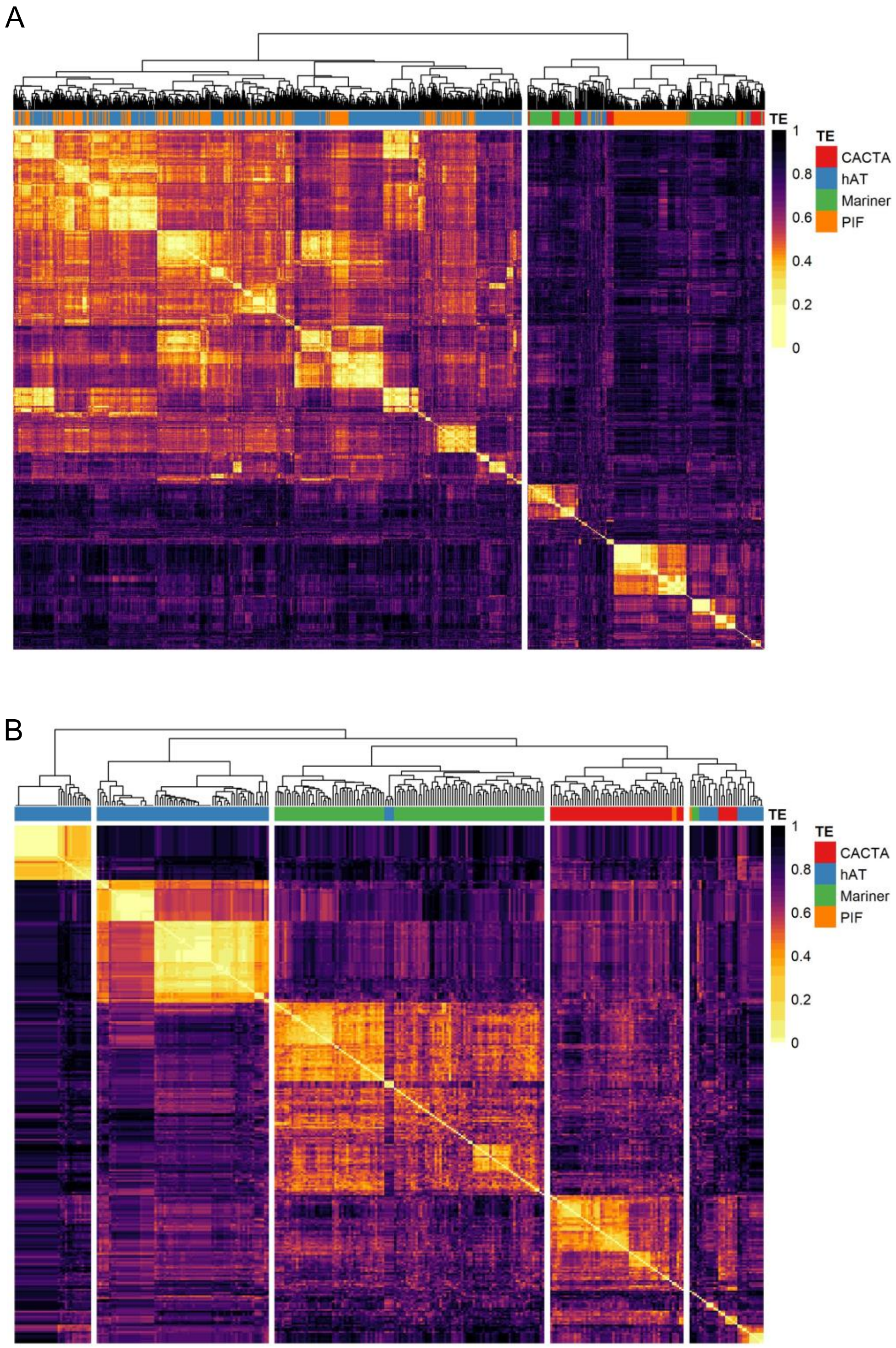

S8 Fig. TIR relationships of TEs annotated using TIRs derived from TIR Learner. Visualisation of the symmetric distance matrix for the forward TIRs of Pack-TYPE TEs annotated using *packFinder* using TIRs derived from TIR Learner; the plot displays a random sample of 1000 of these elements. Distance was calculated using the alignment-free kmer algorithm, and elements were ordered by hierarchical clustering (**Methods**). Two heatmaps were generated for *Zea mays* (**A**) and *Oryza sativa* (**B**).
