# Supplementary material for: The widespread nature of Pack-TYPE transposons reveals their importance for plant genome evolution
Source: PLoS Genet. 2022 Feb 24;18(2):e1010078. doi: 10.1371/journal.pgen.1010078 (PMC8903248; doi:10.1371/journal.pgen.1010078)
Supplement: S9 Fig — (PDF) [file pgen.1010078.s009.pdf]

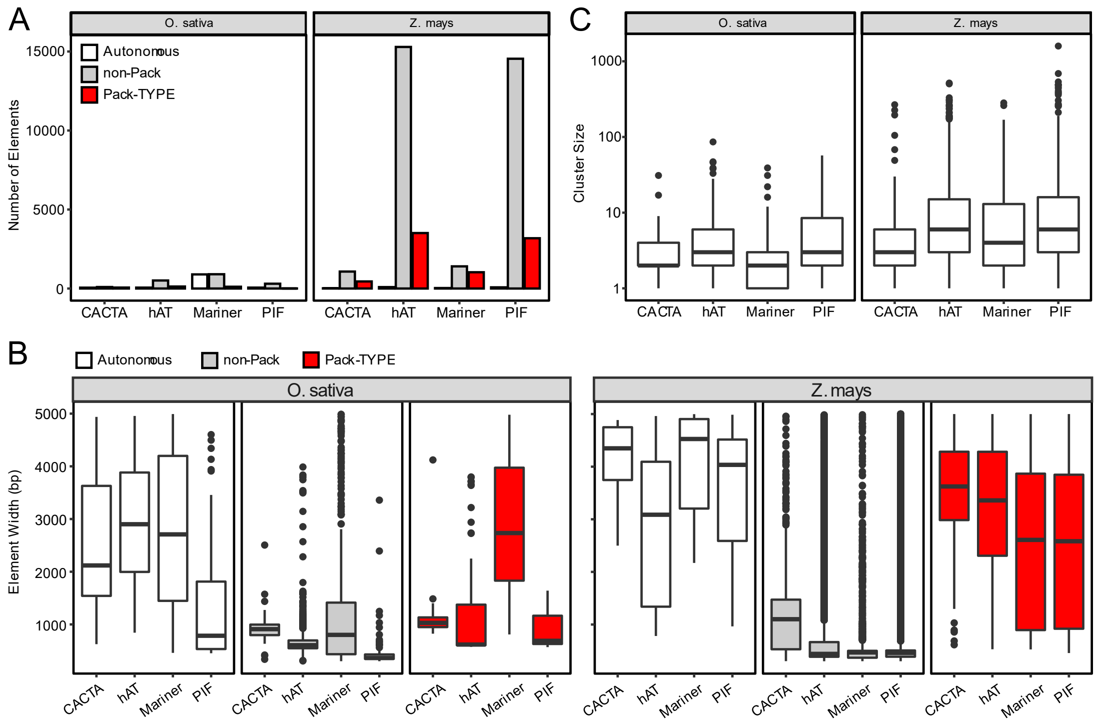


**S9 Fig. The properties of TEs annotated using TIRs derived from TIR Learner. A** Bar plot displaying the total number of elements annotated by *packFinder* in each of the TIR superfamilies tested in both rice (*O. sativa*) and maize (*Z. mays*) genomes. These TIR lists were obtained from TIR Learner. Colours represent TE functional designation, assigned automatically using BLAST. **B** Box plots displaying the distribution of TE widths in each category, defined after the application of the *packFinder* BLAST step in both the rice (*O. sativa*) and maize (*Z. mays*) genomes. **C** Boxplots for the distribution of annotated cluster sizes in both rice (*O. sativa*) and maize (*Z. mays*) genomes.
