# Supplementary material for: The widespread nature of Pack-TYPE transposons reveals their importance for plant genome evolution
Source: PLoS Genet. 2022 Feb 24;18(2):e1010078. doi: 10.1371/journal.pgen.1010078 (PMC8903248; doi:10.1371/journal.pgen.1010078)
Supplement: S11 Fig — (PDF) [file pgen.1010078.s011.pdf]

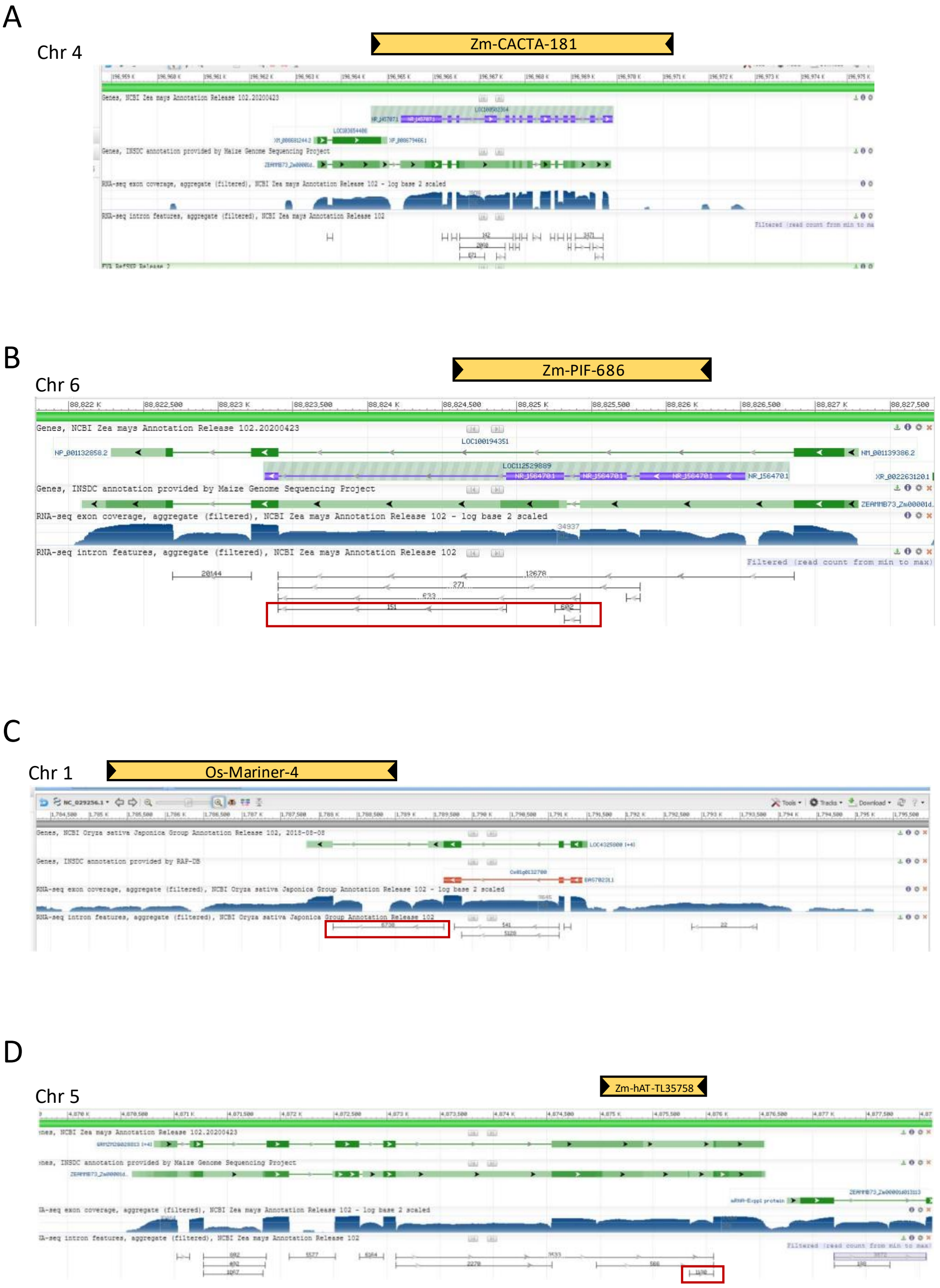

S11 Fig. Expression of Pack-TYPE TEs in maize and rice. Screenshots taken from NCBI Genome Data Viewer browser showing gene annotation and RNA expression from aggregate RNAseq experiments (analysed by NCBI) at specific Pack-TYPE TE example loci illustrated in **Fig 4**, Zm-CACTA-181 (**A**), Zm-PIF-686 (**B**), Os-Mariner-4 (**C**) and Zm-hAT-TL35758 (**D**). The approximative locations of the Pack-TYPE TEs annotated with *packFinder* are represented with a yellow box on top of the tracks. In the bottom track are shown intron splicing events (with supporting RNA-seq reads). With red rectangles are highlighted intron splicing events occurring between RNA transcribed from the Pack-TYPE TE sequences and remaining exons of the same gene, if present.
