# Supplementary material for: The widespread nature of Pack-TYPE transposons reveals their importance for plant genome evolution
Source: PLoS Genet. 2022 Feb 24;18(2):e1010078. doi: 10.1371/journal.pgen.1010078 (PMC8903248; doi:10.1371/journal.pgen.1010078)
Supplement: S10 Fig — (PDF) [file pgen.1010078.s010.pdf]

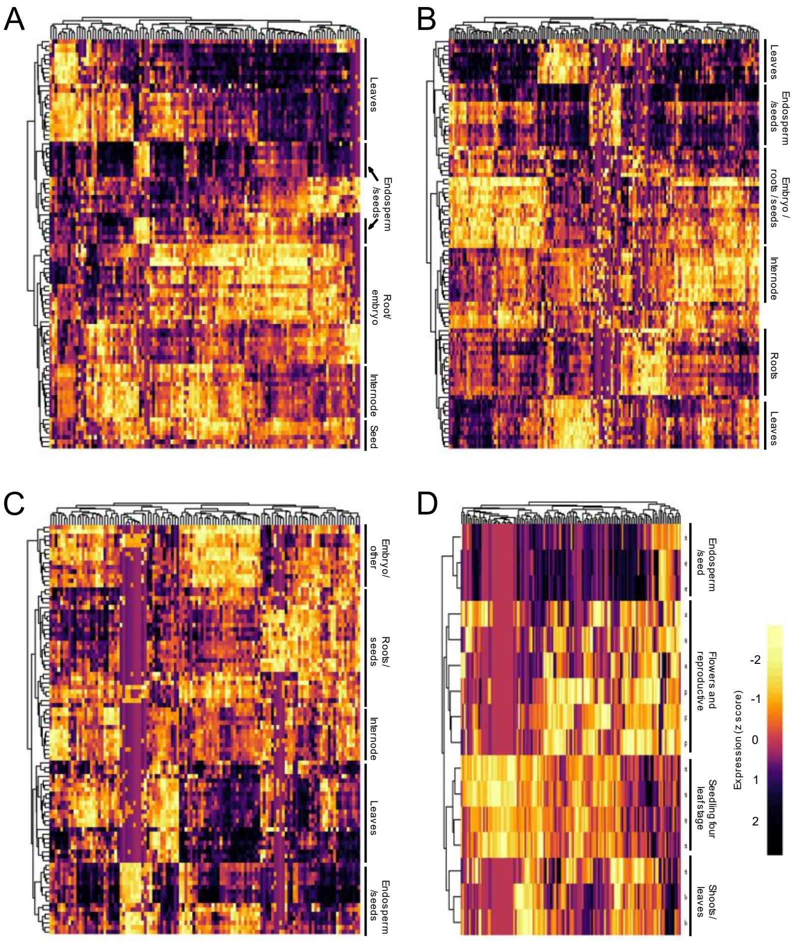

S10 Fig. Tissue-specific expression of genes captured by Pack-TYPE TEs. Heatmaps for the relative expression of genes captured by Pack-TEs; lists were obtained for each TE superfamily and genome, including genes that had significant BLAST matches to Pack-TYPE elements. The rows and columns of the heatmap were ordered by complete-linkage hierarchical clustering. The first three panels represent the genes matching Pack-TEs in maize (**A** - *PIF*, **B** - *hAT*, **C** – *CACTA*). The final panel (**D**) illustrates the expression of genes matching Pack-Mariner elements in rice.
