# Supplementary material for: The widespread nature of Pack-TYPE transposons reveals their importance for plant genome evolution
Source: PLoS Genet. 2022 Feb 24;18(2):e1010078. doi: 10.1371/journal.pgen.1010078 (PMC8903248; doi:10.1371/journal.pgen.1010078)
Supplement: Attachment — Submitted filename: Gisby and Catoni_response to reviewers.pdf [file pgen.1010078.s013.pdf]

Point-to-point answer to referees' comments

We thank all three reviewers for their well-informed and fair comments. We believe their contributions have helped to improve and clarify our manuscript. Please find detailed answers to the reviewers' points marked in red below.

Reviewer #1

The authors mention that "all Pack-TYPEs tested contain less repeated DNA than all TEs annotated in rice and maize belonging to the corresponding superfamily (Figure S3)" on page 9: given the high TE content of the maize genome does this suggest that Pack-TYPE TEs arise in rather gene-rich regions? Apart from MITEs is there a type of class II that has a tendency to insert into gene-rich regions?

Although previous studies found a tendency for Pack-MULEs to insert into gene-rich regions, we did not find a difference in the location of annotated insertions of Pack-TYPEs or non-Pack TEs in the rice and maize genomes. However, the distribution of historical TE insertions in a genome is not only a consequence of preferential insertions, but also the rearrangement of DNA or the positive or negative selection of specific transposition events. Therefore, the study of Pack-TYPE TE mobilisation in real-time is probably the better approach to study preferential insertion. We edited our discussion to comment on this point (page 12 of the revised manuscript).

Out of curiosity I have two more questions:

- Do the authors have evidence that Pack-TYPE TEs display intraspecific polymorphisms ?

This is an interesting point. We observed that several of the identified Pack-TYPE TE clusters are indeed polymorphic, and they tend to include more novel/variable DNA in the internal sequence (Figure 3). However, due to the variability in their sequence, an extensive investigation of such polymorphic nature would require a different approach, and it was not in the scope of this work.

- Are the gene captured expressed ? (for instance from Fig 4?).

To fully address this point we investigated the expression of the genes displayed in Figure 4. The gene models suggest that transcripts are generated by merging exons from Pack-TYPE TEs and the surrounding chromosomal areas. Indeed, in three out of four examples, we observed that these merged transcripts were sequenced in RNA-seq experiments. This result is now illustrated in Figure S10 and described in a paragraph at the end of the Result chapter (page 10).

I wonder if Mariner, hAT etc. should be italicized.

Thank you for the advice; superfamily names have now been italicised.

Page 11: “maize Zm-CACTA-181 TE is located in the 5-prime region of the gene Zm000021d052675”: isn’t it the 3 prime region?

Thank you for noticing this; the text has now been corrected.

Reviewer #2

When run on rice and maize, PACKFinder identified approximately 1,000 and 3,000 TIR elements in these genomes, respectively. These elements are a mix of PACK-type, autonomous, and non-autonomous elements of all major superfamilies of TIR DNA transposons. This number of elements is surprisingly small, especially given that the introduction cites the Jiang et al. 2004 paper, which identifies 3000 Pack-MULEs in rice.

Previous works have shown that Pack-MULEs are exceptionally abundant in rice. When *MULE* TIRs were used as input to *packFinder*, we annotated up to 99% (depending on the parameters used) of the original Pack-MULEs annotated in the rice genome. This entire analysis, which allowed us to evaluate the performance of *packFinder*, is now presented in a dedicated section of the revised manuscript (results described on page 4-5 and methods on page 14).

Then at the bottom of page 14, the authors point to URLs for maize and rice TE annotations that have an order of magnitude more DNA TE elements identified in the respective genomes.

In the cited TE annotation for the maize and rice genomes, TEs have been classified by sequence homology and likely include many incomplete elements. Our tool is designed for functional annotation of intact Pack-TYPE TEs, so we do not expect to find TE remnants or other incomplete elements in our list, and we think it is expected that our Pack-TYPE TE list includes fewer elements compared to public annotations.

In addition, in our original analysis, we used a set of well-characterised TIR sequences as input for *packFinder* to increase confidence in superfamily annotation. We added a new *packFinder* annotation in the revised manuscript, where a more extensive set of maize and rice TIRs have been used (obtained with the tool TIR-Lerner). In this second analysis, we annotated far more TIR TEs (>10,000), suggesting that the input TIR set primarily determines the number of TEs detected.

The authors do not compare PACKFinder TE annotations to the rice or maize annotations, so it is unclear why the number of identified elements is so different. Furthermore, the discussion section describes the benefit of PACKFinder as being a structure-based annotation method rather than homology-based, without comparing to the maize annotation that is already structure based.

While we believe we addressed the difference in the number of annotated elements in the previous point, we also ran a comparison to the maize and rice annotations. Our analysis shows that, similarly to Arabidopsis, the annotation of autonomous TEs generally appeared to be less accurate than the annotation of Pack-TYPE TEs for almost all superfamilies (Figure S3 in the revised version of the manuscript).

In addition to typical considerations for TE annotations, Pack-type TEs require high-quality gene annotations to serve as a reference set to identify gene capture. In the methods, the authors state that Pack-TYPE was defined based on a BLAST hit > 50 bp to a "valid CDS entry". This needs further clarification, especially since 50 bp is so short – quite a bit shorter than the average exon in either rice or maize.

Previous works have set no sequence length requirement for BLAST and have relied solely on the E-value to discriminate Pack-TYPE TEs. We found that, in Arabidopsis, the addition of a length threshold (> 50 bp) did not significantly increase the number of false negatives, and we adopted it for the rest of the analysis. The model describing how Pack-TYPE TEs are generated allows for the acquisition of exon fragments as well as full exons, depending on the locations of two neighbouring Pack-TYPE TE insertions in the same locus. Therefore, we do not believe the use of average exon size as threshold to determine Pack-TYPE TEs is justified.

Additionally, in the revised manuscript, we have included a new annotation performed using an extended set of TIRs obtained from the recently developed tool TIR-Learner (Su et al., 2019) (pages 8-9 of the revised manuscript). Although in this analysis we used a stricter (>250nt length) threshold to define Pack-TYPE TEs, the proprieties of annotated Pack-TYPE-TEs were similar to our first analysis (compare Figure 2 and Figure S8), suggesting that the BLAST thresholds used are not consistently changing the output of the analysis.

I am concerned here because the maize gene annotation used for this study is known for its poor quality, so it is possible that poor annotations could confound results here.

In our pipeline, *packFinder* screens for TEs using structural features (presence of TIRs and TSD) so that their discovery is not affected by previous annotations (except the quality of available TIR sequences). The ability to discriminate between non-Pack, Pack-TYPE and autonomous elements is based on the BLAST tool, so while imprecise coding DNA classification could confuse Pack-TYPE and non-Pack TEs, this will not affect the coordinates of an annotated element.

In fact, I looked at the publicly available maize and rice genome browsers for the four genes shown in figure 4 and for two of them (in 4A and 4B), the alignment of RNA-seq reads does not support the figure. For example, in 4A, only the exons labelled Zm-CACTA-181 are expressed, so TE movement is not necessarily creating a new transcript.

To answer this point, we carefully analysed the expression for all examples shown in Figure 4 (please note we changed the example in 4D), using aggregate transcriptional profiles available from the NCBI website (results displayed in Figure S10 and page 10). While Zm-CACTA-181 (Figure S10A) is expressed in this dataset, we could not find evidence of a new transcript merging the sequence of the TE and the neighbouring exons. However, for the other three examples (Figure S10B, C and D), we found evidence of mRNA molecules including both exons originating from the Pack-TYPE TE and the rest of the locus, supporting the presence of new transcript variants. Therefore, although the reviewer is correct and TE movement is not necessarily creating a new transcript, we found evidence that this is happening at least in three of our four examples, supporting our general conclusion. This analysis has been added to the manuscript (page 10).

It would also be useful to know what kinds of genes are being captured by Pack-TYPE transposons, how many of them have known functions, and how many of them have syntenic orthologs with other grasses.

Thank you for raising this important question. We have investigated the functional enrichment and expression patterns of the original genes from which sequences have been captured by annotated Pack-TYPE elements. We investigated the enrichment of GO terms and used the expression profiles of rice and maize genes to investigate their tissue-specificity. We observed some bias in the types of genes being captured. For instance, “flower development” and “anatomical structure morphogenesis” were enriched in the genes captured by Pack-Mariner TEs in rice. These genes were predominantly expressed in seedlings, flowers, and reproductive tissues. This new analysis is now described on pages 8-10 of the revised manuscript.

Another place where annotation issues could be interfering with the analysis is where the authors find that the median number of distinct loci within a Pack-TYPE transposon in maize is 3. This is very high and it seems improbable that the average element would have moved and picked up that many distinct pieces. I would like to see these captured fragments further analyzed to rule out the possibility that the fragments themselves are repetitive or part of a Mite that has inserted throughout the genome.

We thank the reviewer for this comment. Upon investigation, we realised that our naïve approach was over-estimating the number of distinct loci. Briefly, our new approach does the following: i) order BLAST matches from greatest to lowest coverage; ii) iterate through each match, removing all overlapping BLAST hits and increasing the count of “number of overlapping loci” by one each time. We have manually inspected several elements with captured fragments estimated with the new approach, and we believe the number of distinct loci is now accurate (this is summarised in Table S9 of the revised manuscript).

Overall, while it is valuable to have more bioinformatic tools specific to transposable elements that are easily accessible and easy to run on bioconductor, I would like to see more analysis performed on these annotations.

We are grateful for the reviewer’s valuable suggestions that we believe have improved the quality of our investigation and helped further elucidate the impact of Pack-TEs on genome evolution. We hope that the additional analyses provided in the revised manuscript (described above) can collectively address the reviewer’s points.

Reviewer #3

Furthermore, developing a tool to identify TEs that do not have coding capacity is even more rare – and, importantly, even more difficult. I have my main concerns about this last point, i.e. how can such a tool be efficient with few false positives and at the same time able to identify most of the Pack-TYPE elements in a genome, which I discuss below.
First, however, I am confused with whether this paper is a technical paper or a research paper. Importantly, the pipeline has not been published and peer reviewed before (true?), which may give new ideas and bring up weaknesses that can substantially improve the identification process (this is what I mostly do here!). To my eyes, this is a technical paper, even though it is not set like that, with a little bit of more analysis offered: for example, the 3-4 genes with

synteny, or the example of the internal structure of Packs (3C, 3E). It is not a research paper, as this would require a broader analysis to examine the extent of Pack's in gene evolution, to quantify how many of these cases there are (this is very important, for now we are just looking at some few examples), to analyse if and how the captured or overlapping genes are affected (e.g. expression analysis, to see if they are hypothetical genes or have been annotated with some specific function). For example, I think the title is a bit too much on the biological side for what the paper gives to the reader. I am curious to see what the other reviewers and the editor thinks about that. I believe that some of the above suggestions are not hard to do, but it is not my call.

We are grateful to the reviewer for taking the time to thoroughly review the automated pipeline and provide suggestions for its evaluation. Indeed, we developed the pipeline, and this has not been published before; however, we believe that we also provide significant biological outputs, producing evidence of the existence of Pack-TYPE TEs for all major TIR TEs superfamilies, as well as providing support to the model that describes the biology and evolution of Pack-TYPE TEs. Therefore, we prefer to keep a biological side to our manuscript. Nonetheless, we recognise that the reviewer's request to strengthen the technical side of the manuscript is valid, and we are now providing a set of analyses in the revised version of the manuscript to address these technical points. Please see the specific changes described below.

I think this is a technical paper and it is better if it is developed further in that direction. In this case, some of the Methods can be discussed and analyzed and tested further in Results. A schematic of the pipeline would help.

We produced a new analysis to estimate the performance of *packFinder* (page 5 and see below), and we provided a scheme of the pipeline in Figure 1A.

Figure S2 should become a main. A Table with how many elements of each type were found should be made.

Former Figure S2 has been moved to the main manuscript as Figure 2A. Additionally, a table detailing how many elements of each type has been annotated is included in the revised version as Table S5.

My main concern however is about how well the pipeline works. We have no clear idea about false positives and how many Pack-type we are not finding. I think, based on my experience in this, that it is much safer to use only exons during BLAST; otherwise, one may detect hits with an intron, but if this intron is a TE, then noise and false positives are introduced. 58nt (or 50nt?) is too small. This is the result of the 1e-5 e-value, which is too soft. The distance of up to 15kb between the seed sequences is also too long. My approach would have been stricter.

We agree with the reviewer that further investigation of the pipeline's outputs is required. To answer this point, we performed a new set of analyses. First, we used our tool to reannotate Pack-MULEs in the rice genome. We compared the available Pack-MULE annotation with the *packFinder* outputs, and we estimated true- and false-positive rates for different values of allowable TIR and TSD mismatches (using a distance of up to 15kb). This analysis is described in dedicated paragraphs in Results (page 4-5) and Methods (page 14).

Concerning BLAST, the 50 nt match was selected based on our analysis of Arabidopsis to minimise the number of false negatives, which was then applied to the rice and maize genomes

as well. We have now run a new and more extensive annotation of Pack-TYPE TEs for both species, using as input a more extensive set of TIRs obtained using the TIR-Lerner tool. In this new analysis, we extended the minimum BLAST hit length to 250 nt, and we reduced the maximum size between seeds to 5 kb. The use of an extended set of TIRs allowed us to discover more Pack-TYPE TEs (and better study the expression of captured genes). However, the proportion of Pack-TYPE TEs found, their sizes and relative abundance per superfamily was similar in this analysis and our original output. This suggests that, in the ranges used, the parameters for BLAST and TIR distance are not impacting the overall performance of *packFinder*.

The TIR sequences in Table 1 are sometimes short, and even more so the TSD for Mariners. This is maybe evident in Fig. S2 for Mariners – how do the authors also explain the high k-mer distance in PIFs and CACTA (4th and 5th groups)?

The original organisation of TIR-TE superfamilies was primarily based on similarities in their transposase genes and the structural organisation of representative autonomous elements (which are factors that do not apply to Pack-TYPE TEs). Although TIR sequences tend to be conserved in TEs belonging to the same group, this is not a system that uniquely defines a superfamily. Therefore, from Figure 2A (former Figure S2), it seems that *PIF* and *CACTA* TEs contain subgroups of elements with different TIR sequences (80bp TIR sequences were used for clustering). However, what we want to point out in this figure is that the TIRs of most identified elements are clustering together accordingly to their superfamily (even if in different groups), indicating that the use of a short TIR core and TSD sequence is sufficient for *packFinder* to identify elements that cluster into groups belonging to the same superfamily.

The papers cited in Table 1 for the TIR sequence are old. Are the any newer ones that may better describe these sequences and how the very between species?

The set of TIRs used for our analysis (now in Table S3) was chosen among well-characterised TIR elements in order to precisely assign each pack-TYPE TE to a superfamily, relying only on TIR sequence and TSD (Figure 2B). In the revised version of the manuscript, we annotated more TEs using a new set of TIRs that we extracted from the most recent public TE annotation, using the recently developed tool TIR-Lerner (Su et al., 2019), in both rice and maize genomes. This is described in the revised manuscript on pages 8-9.

All the above are not to say that this pipeline is not good; It can be a valuable addition to the TE toolbox. I am just trying to suggest that more discussion and thinking is needed on the design of the pipeline. Can the authors find a set of well annotated Pack-type TEs in a species and use it as a test to see how well it performs? This will be crucial if such a set exists. I am afraid that, as it is, is largely unknown how well it performs.

Following the reviewer's comment, we performed a new analysis using the Pack-MULE elements, which are well-characterised in the rice genome. We used these Pack-MULEs to compare *packFinder* with the established annotation, and to estimate the specificity and sensitivity of the pipeline according to this reference. This is now described in the revised version of the manuscript (page 4-5 and method section at page 14). We believe that this new analysis supports the good performance of *packFinder* and clarifies the process we employed to select the parameters used in our work.

Some specific comments on the text:

--- 2nd paragraph of Intro 'rearrange host genes'. Sometimes only gene fragments are captured and the original gene remains intact (if capture happened at the RNA level or if it repaired after capture). Sometimes now. I find this confusing, can the authors improve the description here? (same for 'transduplication' in Discussion.

Previous studies show that coding DNA is captured by Pack-TYPE elements at the genomic level (because they also include introns). Following the reviewer's comment, we have clarified this in the revised version of the manuscript (page 3).

--- 2nd paragraph of Results. #40 is not a Pack-type cluster.

Thanks for noticing this, which is due to cluster #40 not passing the threshold for BLAST, resulting in annotation as a non-Pack element. The manuscript text has been modified to consider this point.

--- 2nd paragraph of Results. About non-Pack TEs. How do you know that this is not simply some random genomic sequence? The annotation is non-Pack is based only on the proximity of short sequences (in reverse orientation) and a TSD which can be 2nt in Mariners. This extends to the discussion somewhere else in Results that some non-Pack elements may actually be old Pack-type. This may easily not be the case.

There are several reasons why we believe these are not random sequences:

- In the new analysis performed on Pack-MULEs, we estimated the number of false positives to be less than 1%.
- Specifically for *Mariner* elements, despite their TSD not being highly informative, we still found "TA" as the duplicated terminal sequence in more than 98% of annotated TEs in both maize and rice genomes. Even considering the low CG content of plant genomes, this is far more than expected by chance.
- In our analysis, we removed "singleton" elements under the assumption that TEs are repeated. It is unlikely that random sequences are repeated in the genome.
- While we only used ~10bp TIR sequences to search for TEs, we clustered these elements based on 80 bp of their forward TIR sequences (Figure 2A), demonstrating that TEs belonging to the same superfamily cluster together. Random DNA sequences are not expected to conserve their homology outside the sequence used for their detection.

--- 2nd paragraph of Results. It is too strong to say that the TAIR10 annotation is not reliable. In some cases the Pack pipeline may be wrong, i.e. the gene annotation is correct – we do not know the extent of this.

We agree with the reviewer and have subsequently updated the text in the manuscript to: "This indicates that, compared to autonomous transposons, TAIR10 annotation could be less accurate for Pack-CACTAs or other CACTA-derived non-autonomous elements".

--- Why did the authors not analysed Pack-MULEs as well? This good be a good dataset to compare how the pipeline performs?

We agree with the reviewer that Pack-MULEs represent the best reference set for benchmarking *packFinder*; we have now analysed Pack-MULEs in the rice genome (see above). We used the annotation of Pack-MULEs to evaluate the performance of *packFinder*. This analysis is now described in a dedicated paragraph of the revised version of the manuscript (page 4-5 and page 14).
