# Supplementary material for: The widespread nature of Pack-TYPE transposons reveals their importance for plant genome evolution
Source: PLoS Genet. 2022 Feb 24;18(2):e1010078. doi: 10.1371/journal.pgen.1010078 (PMC8903248; doi:10.1371/journal.pgen.1010078)
Supplement: Attachment — Submitted filename: Gisby and Catoni_response to reviewers_v2.pdf [file pgen.1010078.s014.pdf]

Point-to-point answer to referees' comments

We thank all reviewers for their comments. Please find below answers to the additional points raised by reviewer 2.

Reviewer #2

Thank you for addressing many of my comments in this revision. Many of my major points were addressed and I think that the manuscript was improved with their additional analyses. Specifically, the authors did a nice comparison of the PACKFinder TEs in rice to the existing homology-based annotation. As the authors pointed out, a structure-based annotation typically identifies fewer TEs of higher quality than a sequence homology-based approach.

Thank you for your comment.

However, the authors did not address the differences between their annotation and the maize annotation which is completely different from the rice annotation. The B73v4 TE annotation found at (https://mcstitzer.github.io/maize_TEs/)) is a structure-based TE annotation and still finds many more TEs than PACKFinder. Specifically, the B73v4 TE annotation finds 5,000 hAT, 2,700 CACTA, 63,000 PIF, 900 Mutator, and 66,000 Mariner transposons – far more than PACKFinder. What explaination is there for the widescale differences between PACKfinder and the existing maize annotation? For both maize and rice, what proportion of existing annotations overlap with PACKFinder TEs (like Fig S3 but from the opposite point of view)?

To maximise the confidence in annotation and the quality of the classification of Pack-TYPE elements, we used as input for *packFinder* a limited set of well-known TIR-TEs and the most abundant TIR families for each TE superfamily investigated. In addition, we allowed a minimal amount of TIR/TSD mismatches and removed singleton clusters. Due to the highly conservative nature of this approach, we expect fewer elements to be detected compared to what found in available TE annotations. We calculated the overlap of *packFinder*-detected TEs and the reference annotations in the maize and rice genomes, and these data are illustrated in a new figure (Figure S6A).

The main objective of our analysis was to identify Pack-TYPE TEs in different TE superfamilies and plant genomes, not to provide a new system to generally annotate TIR TEs in plants. The existing TE annotation in maize was produced using multiple methodologies to detect TEs of different superfamilies and classes, and we believe this integrated approach remains the best to comprehensively annotate TEs in this genome.

However, most of structure-based approaches used to annotate TIR TEs in plants use MITEs as model, which do not have additional DNA between TIRs (contrary to Pack-TYPE TEs). For example, in current maize annotation (accordingly to Stitzer et al. 2021), the structural-based search was done with detectMITE tool, with a maximun length limit of 800 bp, which is insufficient to detect most of Pack-TYPE TEs. Indeed, we found that TEs identified with

packFinder tend to be longer compared to TEs found in previous annotations (especially for *Mariner* and *PIF* superfamilies, where MITEs are aboundants) (new Figure S6B and S6C). In addition, maize available annotation does not discriminate between Autonomous, non-Pack and Pack-TYPE elements. For metagenomics analyses, *packFinder* represents a reliable, fast, robust, and easy-to-use tool that annotates Pack-TYPE elements specifically and automatically across different genome references, with little bias towards the quality of previously available TE annotations in a specific species.

Since this is a methods-heavy paper, it would be helpful to include a paragraph breaking down similarities and differences to past annotations in the discussion to explain how this method improves upon prior work in the field.

Following the reviewer's suggestion, we have incorporated the above data and discussion into appropriate sections at pag. 8 and pag. 11-12 (text highlighted in yellow) in the revised manuscript, to clarify how *packFinder* can improve the annotation of Pack-TYPE TEs.
